# Cell Death Patterns Due to Warm Ischemia or Reperfusion in Renal Tubular Epithelial Cells Originating from Human, Mouse, or the Native Hibernator Hamster

**DOI:** 10.3390/biology7040048

**Published:** 2018-11-15

**Authors:** Theodoros Eleftheriadis, Georgios Pissas, Georgia Antoniadi, Vassilios Liakopoulos, Ioannis Stefanidis

**Affiliations:** Department of Nephrology, Faculty of Medicine, University of Thessaly, 41110 Larissa, Greece; gpissas@msn.com (G.P.); g.antoniadi@yahoo.com (G.A.); liakopul@otenet.gr (V.L.); stefanid@med.uth.gr (I.S.)

**Keywords:** ischemia–reperfusion, hibernation, apoptosis, autophagy, necroptosis, lipid peroxidation, ferroptosis

## Abstract

Ischemia–reperfusion injury contributes to the pathogenesis of many diseases, with acute kidney injury included. Hibernating mammals survive prolonged bouts of deep torpor with a dramatic drop in blood pressure, heart, and breathing rates, interspersed with short periods of arousal and, consequently, ischemia–reperfusion injury. Clarifying the differences under warm anoxia or reoxygenation between human cells and cells from a native hibernator may reveal interventions for rendering human cells resistant to ischemia–reperfusion injury. Human and hamster renal proximal tubular epithelial cells (RPTECs) were cultured under warm anoxia or reoxygenation. Mouse RPTECs were used as a phylogenetic control for hamster cells. Cell death was assessed by both cell imaging and lactate dehydrogenase (LDH) release assay, apoptosis by cleaved caspase-3, autophagy by microtubule-associated protein 1-light chain 3 B II (LC3B-II) to LC3B-I ratio, necroptosis by phosphorylated mixed-lineage kinase domain-like pseudokinase, reactive oxygen species (ROS) fluorometrically, and lipid peroxidation, the end-point of ferroptosis, by malondialdehyde. Human cells died after short periods of warm anoxia or reoxygenation, whereas hamster cells were extremely resistant. In human cells, apoptosis contributed to cell death under both anoxia and reoxygenation. Although under reoxygenation, ROS increased in both human and hamster RPTECs, lipid peroxidation-induced cell death was detected only in human cells. Autophagy was observed only in human cells under both conditions. Necroptosis was not detected in any of the evaluated cells. Clarifying the ways that are responsible for hamster RPTECs escaping from apoptosis and lipid peroxidation-induced cell death may reveal interventions for preventing ischemia–reperfusion-induced acute kidney injury in humans.

## 1. Introduction

Decreased blood perfusion induces organ dysfunction due to the lack of oxygen and nutrients, resulting in the disruption of oxidative phosphorylation and energy collapse of the cells. Reperfusion may also result in cell death due to the massive re-entry of nutrients and, especially, oxygen that leads to the production of large amounts of reactive oxygen species (ROS) [1,2]. In addition to cardiovascular diseases caused by an obstructed artery, the same events also take place under conditions of impaired effective blood volume, as in various forms of shock, often leading to multiorgan failure and death [3]. The kidney is particularly sensitive to the reduction in blood flow, since the partial pressure of oxygen is relatively low in this organ, and the renal tubular epithelial cells have high energy demands for maintaining organism homeostasis [4,5]. 

Comparative biology could yield new and possibly clinically useful information for the prevention or treatment of ischemia–reperfusion (I-R) injury. To cope with the food shortage during the winter months, many mammals fall into a state of hibernation. In this situation, they survive for months by decreasing their metabolic rate. Interestingly, hibernation encompasses prolonged bouts of deep torpor with a dramatic fall in body temperature, and heart and breathing rates, resulting in an ischemic state of the whole organism. These long bouts of deep torpor are interspersed by short periods of arousal when animals rewarm themselves back to euthermia for several hours. During arousals, the heart rhythm and the number of breaths increase, setting the organism in a state of reperfusion [6,7]. However, these animals survive, showing no evidence of I-R injury in the brain, heart, kidneys, or other organs. For instance, the kidneys of the dormouse *Muscardinus avellanarius* preserve kidney ultrastructure during hibernation and arousal from hibernation [8], and the Syrian hamster kidneys do not exhibit significant functional or pathologic changes after induction of torpor [9]. 

Most animals that fall into winter hibernation lower their metabolic rate and, consequently, their body temperature. Therefore, they are characterized by cold I-R. The latter can act protectively, although during mid-arousals, the body temperature rises to normal levels [7,10]. However, these animals are more resistant to warm I-R injury than phylogenetically related species that cannot hibernate [11,12]. Regarding the kidneys, although no direct ischemia–reperfusion studies by clamping renal artery/vein have been performed in hibernators, cardiac arrest, or hemorrhagic shock followed by resuscitation, incidents that correspond to warm I-R induce significant functional renal impairment and pathological damage in rats, while arctic ground squirrels are protected [13]. Such experimental results question the role of low body temperature or winter season in I-R injury resistance in hibernators [14]. Interestingly, two species of mouse-tailed bats fall into hibernation in the high ambient temperature of geothermal caves without a significant drop in their body temperature [15]. Also, the fact that certain primates phylogenetically close to humans, such as *Cheirogaleus medius* (fat-tailed dwarf lemur), fall into hibernation while maintaining a relatively high body temperature [16,17], makes it possible that human cells may be able to demonstrate resistance to warm I-R injury after some intervention.

This study aimed to compare the resistance to warm ischemia, and followed up reperfusion of cells originating from human and the native hibernator, *Mesocricetus auratus* (Syrian hamster). Cells from *Mus musculus* (mouse), a rodent that does not hibernate, were used as a phylogenetic control group for Syrian hamster cells. Renal proximal tubular epithelial cells derived from these species were selected for the study, as the kidney is an I-R sensitive organ, and the particular area of the kidney that is the most vulnerable due to the high metabolic demands of the above cells [4,5]. The different types of warm ischemia or reperfusion-induced cell death were also evaluated. Even though I-R injury has been studied extensively in human and mouse renal tubular epithelial cells, there is controversy as to whether the cell death is due to apoptosis, autophagy, or various types of regulated cell necrosis, such as necroptosis or lipid peroxidation-induced cell death [18,19,20,21,22]. Membrane lipid peroxidation, by disrupting cell membrane function, induces cell death [21], and ferroptosis is such a kind of death [22]. Clarifying the differences in cell death patterns due to warm anoxia and reoxygenation between human cells and cells from a native hibernator, and understanding how hibernators cope with these detrimental conditions, may reveal new interventions for rendering human cells more resistant to I-R injury.

## 2. Materials and Methods

### 2.1. Cell Culture, Treatment, and Imaging

Primary human renal proximal tubular epithelial cells (RPTECs) (cat. no. 4100, ScienCell, Carlsbad, CA, USA), primary Syrian hamster RPTECs (cat. no. HM-6015, Cell Biologics, Chicago, IL, USA) and primary C57BL/6 mouse RPTECs (cat. no. C57-6015, Cell Biologics) were cultured in Complete Epithelial Cell Medium/w kit (cat. no. M6621, Cell Biologics), supplemented with epithelial cell growth supplement, antibiotics, and fetal bovine serum. All the above primary cells were differentiated, well-characterized passage one RPTECs. We expanded them in 75 cm^2^ flasks and, consequently, passage two cells were used for the experiments.

Cells were cultured in 6-well plates at a number of 300,000 cells per well, or in 96-well plates at a number of 10,000 cells per well, for 16 h, before the onset of anoxic conditions. The confluency of the cells, as estimated by inverted microscopy, did not differ at the start of each experiment. The GasPak^TM^ EZ Anaerobe Container System with Indicator (cat. no. 26001, BD Biosciences, S. Plainfield, NJ, USA) was used to reduce oxygen levels to less than 1%. Cells within the anaerobe container were cultured at 37 °C. These anoxic conditions imitate warm ischemia.

Cell photos were captured at the onset of hypoxia and at 2-h intervals. For this purpose, an inverted microscope (Axiovert 40C, Carl Zeiss Light Microscopy, Göttingen, Germany) and a digital camera with the related software (3MP USB2.0 Microscope Digital Camera, Amscope, Irvine, CA, USA) were used. 

Imaging of each cell type was used to detect the approximate time of severe cell deterioration (death) due to anoxia. Reperfusion experiments were started at a point corresponding to half of this time. In these experiments, cells were washed, supplemented with fresh culture medium, and placed at 37 °C in a humidified atmosphere containing 5% CO_2_. These reoxygenation conditions imitate warm reperfusion. The time point of severe cell deterioration due to reoxygenation was also detected with imaging.

As live cells were required for conducting the experiments, the various parameters of the study were evaluated at the halfway point of the time needed for detecting severe cell deterioration, with cell imaging under anoxia or after 2 h of reoxygenation. The same time points for mouse and hamster cells were used, since the latter showed remarkable resistance to cell death by anoxia. All the experiments were performed 9 times.

### 2.2. Biochemical Assessment of Cell Death

Since cell imaging can provide information about the general state of the cells, but not in a strictly quantitative manner, cell death was also assessed biochemically. Cells were cultured in 96-well plates, as described above. Cell death was assessed by LDH release assay using the Cytotox Non-Radioactive Cytotoxic Assay kit (cat. no. G1780, Promega Corporation, Madison, WI, USA). 

### 2.3. Assessment of Cell Apoptosis

Cell apoptosis was assessed by the level of activated cleaved caspase-3. Cells were cultured in 6-well plates as described above. Cells were lysed using the T-PER (tissue protein extraction reagent) (Thermo Fisher Scientific Inc., Rockford, IL, USA) supplemented with protease and phosphatase inhibitors (Sigma-Aldrich; Merck Millipore Darmstadt, Germany and Roche Diagnostics, Indianapolis, IN, USA, respectively). Protein was quantified via Bradford assay (Sigma-Aldrich; Merck Millipore) and 10 μg from each sample was used for Western blotting. Blots were incubated with the primary antibody against cleaved caspase-3 for 16 h at 4 °C, followed by incubation of the secondary antibody (anti-rabbit IgG, horseradish peroxidase (HRP)-linked Antibody, cat. no. 7074, Cell Signaling Technology, Danvers, MA, USA) for 30 min at room temperature. The primary antibody against cleaved caspase-3 for primary human RPTECs was a rabbit monoclonal antibody purchased from Cell Signaling Technology (cat. no. 9664), whereas the one for hamster and mouse RPTECs was a rabbit polyclonal antibody from Abcam, Cambridge, UK (cat. no. ab13847). Cleaved caspase-3 expression was normalized to the expression of β-actin (cat. no. 4967, Cell Signaling Technology). In the case of reprobing polyvinylidene fluoride (PVDF) blots, the previous primary and secondary antibody were removed with the Restore Western Blot Stripping Buffer (Thermo Fisher Scientific Inc.) according to the manufacturer’s protocol. Analysis of the Western blots was performed using ImageJ software (National Institute of Health, Bethesda, MD, USA) [23].

In parallel with cleaved caspase-3, the expression of the tumor suppressor p53, a major pro-apoptotic factor, was evaluated using a rabbit polyclonal antibody (cat. no. 9282, Cell Signaling Technology) for human RPTECs, and a mouse monoclonal antibody (cat. no. 2524, Cell Signaling Technology) for mouse and hamster RPTECs. For the last, an anti-mouse IgG, HRP-linked antibody was used as a secondary antibody (cat. no. 7076, Cell Signaling Technology).

### 2.4. Assessment of Cell Autophagy

Autophagy was assessed by the phosphatidylethanolamine-conjugated microtubule-associated protein 1-light chain 3 B II (LC3B-II) to unconjugated LC3B-I ratio. LC3B-I and LC3B-II expression were evaluated using Western blotting, as described above. For this purpose, an anti-LC3B rabbit polyclonal antibody was used (cat. no. ab48394, Abcam).

### 2.5. Assessment of Cell Necroptosis

Cell necroptosis was evaluated by the level of phosphorylated mixed-lineage kinase domain-like pseudokinase (p-MLKL), assessed using Western blotting, as described above. The anti-MLKL (phospho S358) rabbit monoclonal antibody [EPR9514] (cat. no. ab187091, Abcam) was used for human RPTECs, whereas the anti-MLKL (phospho S345) rabbit monoclonal antibody (EPR9515(2)) (cat. no. ab196436, Abcam) was used for hamster and mouse RPTECs.

### 2.6. Assessment of Reactive Oxygen Species Production and Lipid Peroxidation

For assessing ROS production, cells were cultured in 96-well plates. Once the incubation period was over, the cells were stained with 5 μM of the fluorogenic probe CellROX^®^ Deep Red Reagent (cat. no. C10422, Invitrogen, Life Technologies, Carlsbad, CA, USA) for 30 min at 37 °C. The cells were then washed with phosphate-buffered saline (PBS), and fluorescence signal intensity was measured and analyzed on an EnSpire^®^ Multimode Plate Reader (Perkin Elmer, Waltham, MA, USA).

For assessing lipid peroxidation, cells were cultured in 6-well plates. The end product of lipid peroxidation malondialdehyde (MDA) was measured fluorometrically in cell extracts using the Lipid Peroxidation (MDA) Assay Kit (cat. no. ab118970, Abcam). This assay detects MDA levels as low as 1 nmol/well via colorimetry, and 0.1 nmol/well through fluorometry. Before MDA measurement, Bradford assay was performed, and the lysate volume of each sample was adjusted to hold an equal protein concentration.

### 2.7. Statistical Analysis

The normality of the evaluated variables was assessed and confirmed by one-sample Kolmogorov–Smirnov test. For comparison of means, a one-way analysis of variance (ANOVA) followed by Bonferroni’s correction test was used. Especially for statistical analysis of the cell imaging results, the Kruskal–Wallis H test was used since these results did not follow the normal distribution. Results were expressed as mean ± SD, and a *p* < 0.05 was considered statistically significant. For the reader’s convenience, the results are shown after normalization of means for the control group of each cell type. Statistical analysis was performed with the IBM SPSS Statistics for Windows, Version 20 (IBM Corp., Armonk, NY, USA). 

## 3. Results

### 3.1. Sensitivity of Human, Mouse, and Hamster RPTECs to Death Due to Anoxia or Reoxygenation

**Figure 1:**
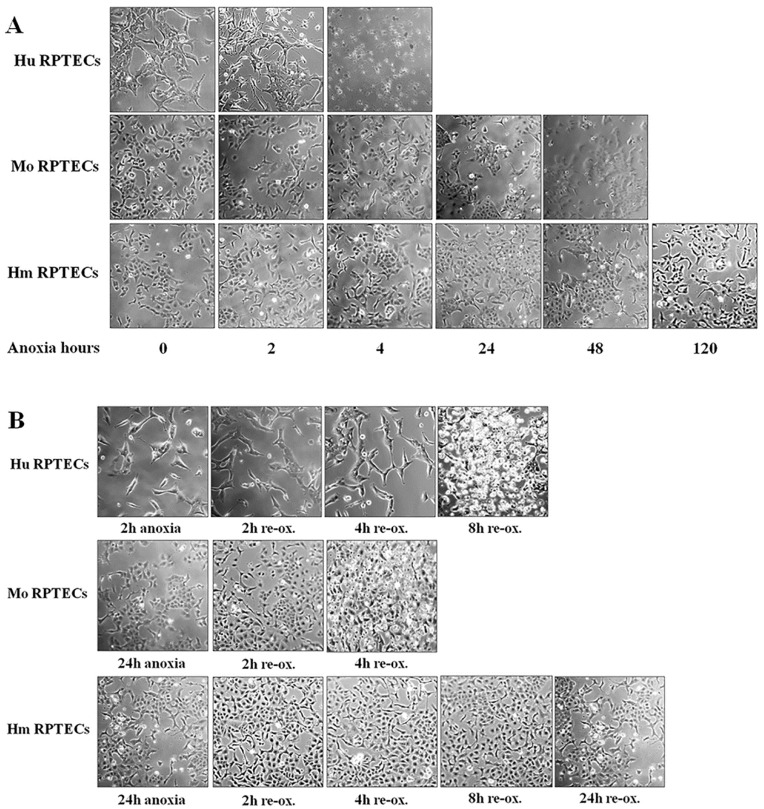
**Sensitivity of human, mouse, and hamster renal proximal tubular epithelial cells (RPTECs) to death due to anoxia or reoxygenation**. Human RPTECs were extremely sensitive to anoxia since they died after 4 h of anoxia. Mouse RPTECs were less sensitive than human RPTECs, since they died after 48 h of anoxia. On the contrary, hamster RPTECs were extremely resistant to anoxic conditions, since they remained alive even after 120 h of observation (A). During reoxygenation, human RPTECs deteriorated significantly, showing condensation and loss of adherence on a very large scale, after 8 h. Mouse RPTECs were even more sensitive to reoxygenation, since they deteriorated considerably after 4 h. On the contrary, hamster RPTECs were extremely resistant to reoxygenation, since they preserved their morphology after 48 h of observation (B). The photos are representative of one of the nine performed experiments. Hu: human; Mo: mouse; Hm: hamster.

Cell imaging revealed that human RPTECs were extremely sensitive to anoxia, since they died after 4 h of anoxia. Mouse RPTECs were less sensitive than human RPTECs, since they died after 48 h of anoxia (*p* < 0.001). On the contrary, hamster RPTECs were extremely resistant to anoxic conditions, since they remained alive even after 120 h of observation (*p* < 0.001) (Figure 1A).

Cell imaging revealed that human RPTECs deteriorated significantly, showing condensation and loss of adherence on a very large scale, after 8 h of reoxygenation. Mouse RPTECs were even more sensitive to reoxygenation since they deteriorated substantially after 4 h of reoxygenation (*p* < 0.001). On the contrary, hamster RPTECs were extremely resistant to reoxygenation since they retained their morphology after 48 h of observation (*p* < 0.001) (Figure 1B).

**Figure 2:**
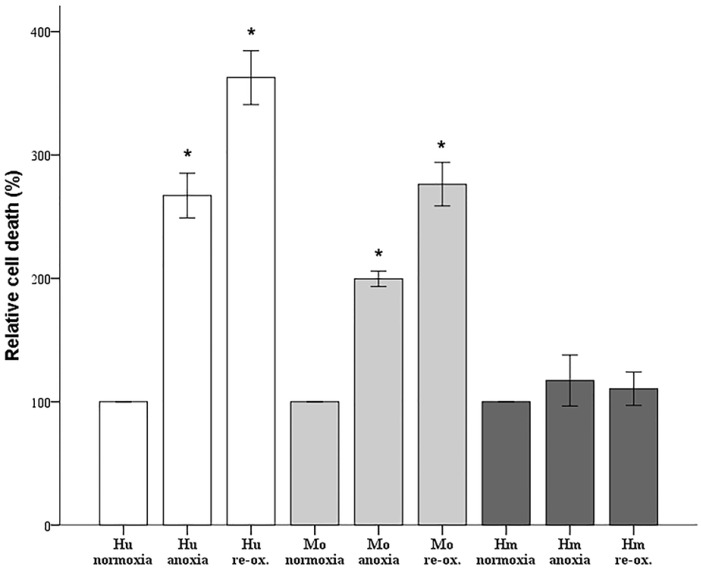
**Biochemical detection of cell death in human, mouse, and hamster RPTECs under anoxia or reoxygenation.** Compared to the control conditions, both anoxia and reoxygenation increased cell death in human and mouse RPTECs. On the contrary, neither anoxia nor reoxygenation affected cell death significantly in hamster RPTECs. Hu: human; Mo: mouse; Hm: hamster. Asterisk indicates a *p* < 0.05 compared to the control cultured under normoxia cells, and error bars correspond to SD.

Cell imaging results were recapitulated by the LDH release assay, which detects both necrotic and apoptotic cell death [24]. LDH release assay was performed at the halfway point of the time required for severe cell deterioration due to anoxia or 2 h after reoxygenation. Compared to control conditions, anoxia increased cell death in human RPTECs by 267 ± 18% (*p* < 0.001) and reoxygenation by 363 ± 22% (*p* < 0.0001). In mouse RPTECs, anoxia increases cell death by 200 ± 6% (*p* < 0.001) and reoxygenation by 276 ± 18% (*p* < 0.001). On the contrary, neither anoxia nor reoxygenation induced cell death in hamster RPTECs. Compared to the control group, cell death assessed by LDH release assay was at 117 ± 21% under anoxic conditions (*p* = 0.06), and at 111 ± 14% after reoxygenation (*p* = 0.385) (Figure 2).

### 3.2. Apoptotic Human, Mouse, and Hamster RPTEC Death Due to Anoxia or Reoxygenation

**Figure 3:**
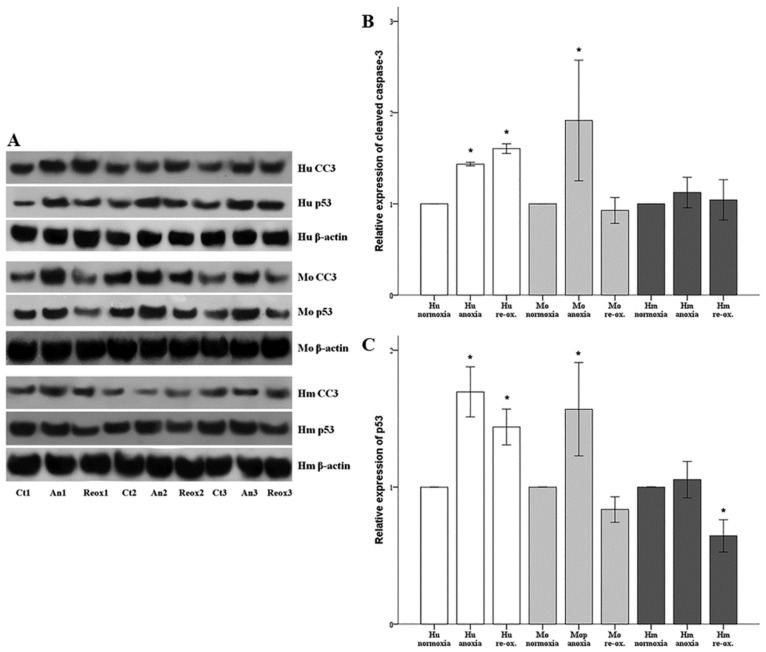
**Apoptosis and expression of the pro-apoptotic p53 in human, mouse, and hamster RPTECs under anoxia or reoxygenation.** Apoptosis was assessed by the level of cleaved caspase-3. Both cleaved caspase-3 and the pro-apoptotic p53 were measured by Western blotting and the results of three, representative of the nine performed experiments, are depicted in panel (**A**). Compared to the control group, in human RPTECs, both anoxia and reoxygenation increased the level of cleaved caspase-3. In mouse RPTECs, cleaved caspase-3 increased only under anoxia, whereas, in hamster RPTECs, apoptosis was not observed either under anoxia or reoxygenation (**B**). The alterations in cleaved caspase-3 fitted to the alterations of the pro-apoptotic protein tumor suppressor p53 (**C**). Hu: human; Mo: mouse; and Hm: hamster. Asterisk indicates a *p* < 0.05 compared to the control cultured under normoxia cells, and error bars correspond to SD.

Apoptosis was assessed by the level of cleaved caspase-3 in cells cultured under anoxic conditions or after reoxygenation at the designated time points in all three cell lines. Compared to the control group, in human RPTECs anoxia increased the level of cleaved caspase-3 by a factor of 1.44 ± 0.02 (*p* < 0.001), and reoxygenation by a factor of 1.61 ± 0.05 (*p* < 0.001). Thus, in human RPTECs, apoptosis occurs both during anoxia and reoxygenation. In mouse RPTECs, anoxia increased cleaved caspase-3 by a factor of 1.91 ± 0.66 (*p* < 0.001), but reoxygenation did not affect its level significantly, since it was 0.93 ± 0.14 of the control (*p* = 1.0). Thus, in mouse RPTECs, apoptosis takes place during anoxia, but not during reoxygenation. In hamster RPTECs, apoptosis was not observed either under anoxia or reoxygenation. Under anoxia, cleaved caspase-3 level was 1.12 ± 0.17-fold the level of the control group (*p* = 0.334) and, after 2 h of reoxygenation, at 1.04 ± 0.16 of the control (*p* = 1.0) (Figure 3A,B). 

The alterations in the expression of cleaved caspase-3 were unanimous with the changes in the expression of pro-apoptotic protein tumor suppressor p53. Compared to the control of human RPTECs group, anoxia increased the level of p53 by a factor of 1.70 ± 0.18 (*p* < 0.001), and reoxygenation by a factor of 1.44 ± 0.13 (*p* < 0.001). In mouse RPTECs, anoxia increased p53 by a factor of 1.57 ± 0.66 (*p* < 0.001), yet reoxygenation did not affect its level significantly, as it was at 0.84 ± 0.09 of the control (*p* = 0.304). In hamster RPTECs, the p53 level was not increased either under anoxia or reoxygenation. Under anoxia, p53 expression was almost the same as the one found in the control group (1.05 ± 0.13, *p* = 0.808), whereas, after 2 h of reoxygenation, p53 level decreased at the 0.64 ± 0.12 of the control (*p* < 0.001) (Figure 3A,C).

### 3.3. Autophagy in Human, Mouse, and Hamster RPTECs under Anoxia or Reoxygenation

**Figure 4:**
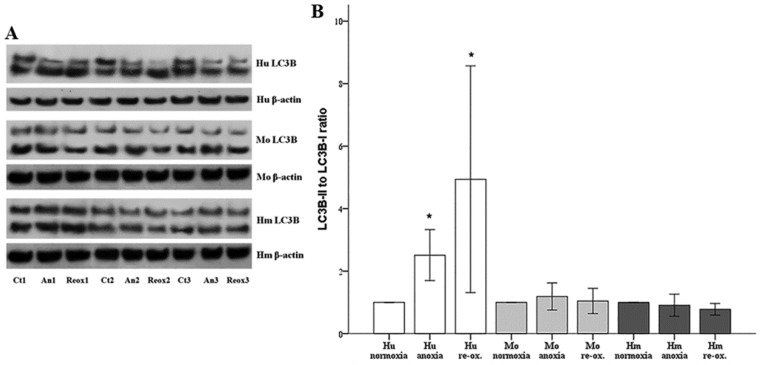
**Autophagy in human, mouse, and hamster RPTECs under anoxia or reoxygenation.** Autophagy was evaluated by the LC3B-II to LC3B-I ratio, assessed by Western blotting. In panel (**A**) three representatives of the nine performed experiments are depicted. Autophagy was observed only in human RPTECs under both anoxia and reoxygenation. Neither in mouse nor in hamster RPTECs did anoxia or reoxygenation induce autophagy (**B**). Hu: human; Mo: mouse; and Hm: hamster. Asterisk indicates a *p* < 0.05 compared to the control cultured under normoxia cells, and error bars correspond to SD.

Autophagy was assessed by the LC3B-II to LC3B-I ratio. Cells were cultured under the same anoxic and reoxygenation chronic periods, as previously described. Both anoxia and reoxygenation induced autophagy in human RPTECs. Compared to the control, the LC3B-II to LC3B-I ratio was increased 2.51 ± 0.82-fold due to anoxia (*p* = 0.001), and 4.94 ± 3.63-fold following 2 h of reoxygenation (*p* = 0.012). Autophagy was not observed under anoxia (LC3B-II to LC3B-I ratio 1.19 ± 0.43 of the control, *p* = 0.751) or reoxygenation (LC3B-II to LC3B-I ratio 1.05 ± 0.41 of the control, *p* = 1.0) in mouse RPTECs. Similarly, in hamster RPTECs, neither anoxia nor reoxygenation induced autophagy since, under anoxia, the LC3B-II to LC3B-I ratio was 0.91 ± 0.35-fold of the control (*p* = 1.0) and, after reoxygenation, the ratio was 0.78 ± 0.19-fold of the control (*p* = 0.160) (Figure 4A,B).

### 3.4. Necroptosis in Human, Mouse, and Hamster RPTECs under Anoxia or Reoxygenation

**Figure 5:**
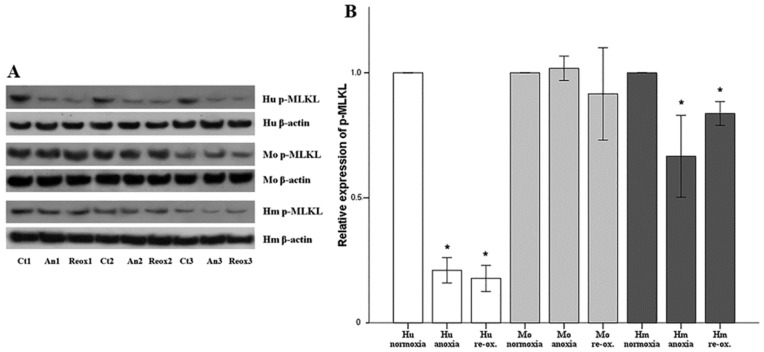
**Necroptosis in human, mouse, and hamster RPTECs under anoxia or reoxygenation.** Necroptosis was evaluated by the level of p-MLKL assessed by Western blotting. In panel (**A**), three representatives of the nine performed experiments are depicted. Necroptosis was not observed in any of the evaluated cells under anoxia or reoxygenation. Phosphorylated MLKL decreased in human and hamster RPTECs under both conditions, and remained unaltered in mouse RPTECs (**B**). Hu: human; Mo: mouse; and Hm: hamster. Asterisk indicates a *p* < 0.05 compared to the control cultured under normoxia cells, and error bars correspond to SD.

Cell necroptosis can be evidenced by the level of p-MLKL. Neither anoxia nor reoxygenation induced cell necroptosis in human, mouse, or hamster RPTECs, since p-MLKL was not increased in any case. In human RPTECs, anoxia decreased p-MLKL at 0.21 ± 0.05 of the control level (*p* < 0.001), and reoxygenation at 0.18 ± 0.05 of the control level (*p* < 0.001). In mouse RPTECs, neither anoxia nor reoxygenation altered p-MLKL levels significantly. In the case of anoxia, its level was 1.02 ± 0.05-fold of the control (*p* = 1.0) and, in the case of reoxygenation, 0.92 ± 0.18-fold of the control (*p* = 0.351). Finally, in hamster RPTECs, anoxia decreased p-MLKL at 0.67 ± 0.16 of the control level (*p* < 0.001), and reoxygenation at the 0.84 ± 0.05 of the control level (*p* = 0.005) (Figure 5). 

### 3.5. ROS Production and Lipid Peroxidation in Human, Mouse, and Hamster RPTECs under Anoxia or Reoxygenation

**Figure 6:**
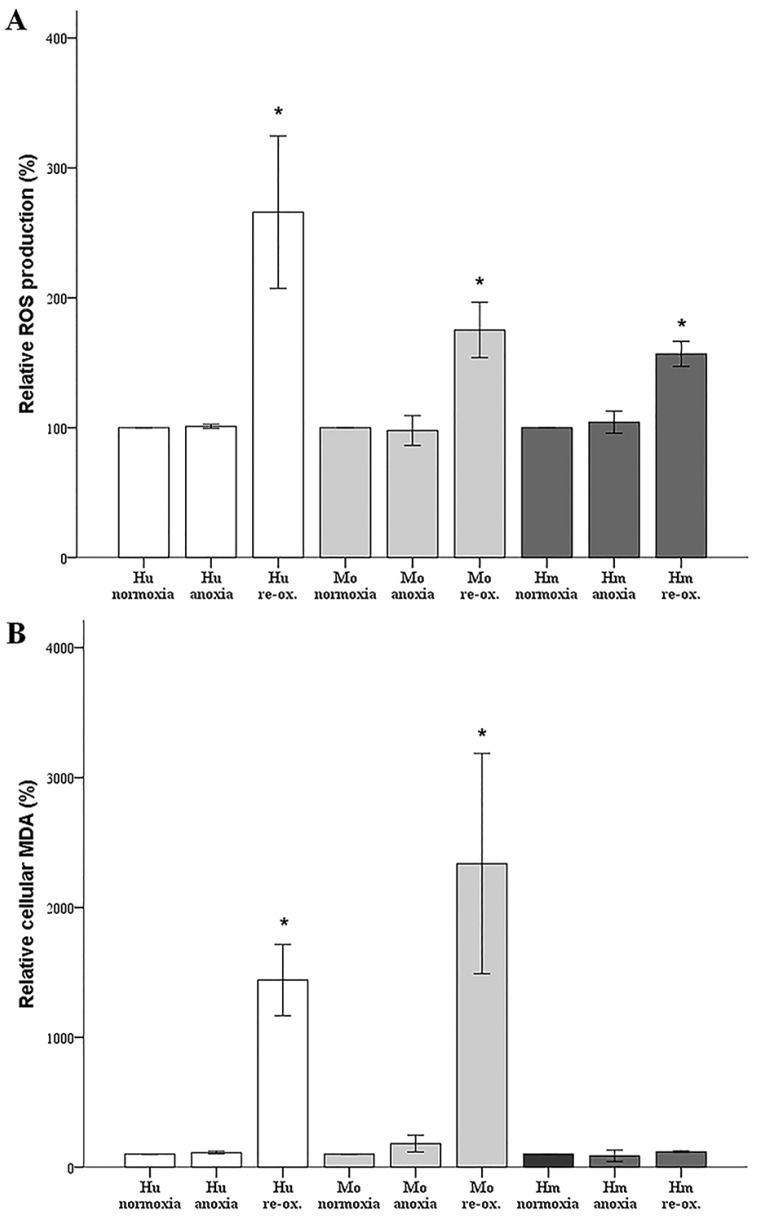
**Reactive oxygen species production and lipid peroxidation in human, mouse, and hamster RPTECs under anoxia or reoxygenation**. Anoxia did not alter ROS in any of the evaluated cells, whereas reoxygenation increased ROS in all of them (A). Lipid peroxidation, a known mechanism of cell death, was assessed by the level of cellular malondialdehyde (MDA). Anoxia did not affect MDA in any of the evaluated cells. Reoxygenation induced a dramatic increase in MDA level in human and mouse RPTECs, but it did not alter the MDA level in hamster RPTECs (B). Hu: human; Mo: mouse; and Hm: hamster. Asterisk indicates a *p* < 0.05 compared to the control cultured under normoxia cells, and error bars correspond to SD.

ROS production and lipid peroxidation, a potential mechanism of cell death [21], and also the end-point of ferroptosis [22], were also assessed during the same experimental process. Anoxia did not affect the ROS level of human RPTECs, since it was found to be at 101 ± 16% of the value found in control RPTECs (*p* = 1.0). Reoxygenation increased ROS to 266 ± 59% of the control (*p* < 0.001). Anoxia did not affect the ROS level in mouse RPTECs as well (98 ± 11% of the control, *p* = 1.0), whereas reoxygenation increased the level of ROS to 175 ± 21% of the control (*p* < 0.001). Similarly, in hamster RPTECs, anoxia did not affect the ROS level significantly since it was found to be at 104 ± 9% of the value found in control RPTECs (*p* = 0.687). Reoxygenation increased ROS level to 157 ± 10% of the control (*p* < 0.001). Hence, in the RPTECs of all evaluated species, anoxia did not affect ROS production, but reoxygenation increased it significantly (Figure 6A).

Anoxia did not affect the MDA level in human RPTECs (112 ± 10% of the control, *p* = 1.0), whereas reoxygenation increased the MDA level robustly to 1441 ± 275% of the control (*p* < 0.001). Also, in mouse RPTECs, anoxia did not alter the MDA level significantly, since it was at 181 ± 64% of the value found in control RPTECs (*p* = 1.0), while reoxygenation markedly increased MDA by 2339 ± 848% (*p* = 0.001). In hamster RPTECs, anoxia did not alter the MDA level significantly (87 ± 44% of the control, *p* = 0.850). However, and contrary to the other evaluated RPTECs, reoxygenation did not affect MDA significantly in hamster RPTECs, since its level was at the 119 ± 5% of the control (*p* = 0.389) (Figure 6B). 

## 4. Discussion

This study aimed to evaluate the differences in resistance to warm ischemia and reperfusion, and the patterns of cell death between human and natural hibernator Syrian hamster RPTECs. Research on the field of hibernation may give rise to new methods in preventing I-R injury, which may prove useful in organ preservation [25,26], as well as in various diseases, such as acute myocardial infarction [1], cerebral ischemia [2], multiple organ failure [3], and ischemic acute kidney injury (AKI) [4,5]. 

Firstly, we evaluated the differences in resistance to ischemia or reperfusion of the RPTECs from the different species, both by cell imaging and biochemically. For this purpose, we used commercially available, well characterized, passage one primary cells, instead of cell lines, in order to obtain more reliable results, since cell lines bear many genetic defects. For instance, the human embryonic kidney 293 (HEK293) cell line, which is commonly used in I-R studies, is characterized, among others, by the lack of a functional p53 [27]. Human RPTECs were very sensitive to both warm anoxia and reoxygenation, while hamster RPTECs were not affected by either anoxia or reoxygenation. Interestingly, like human RPTECs, mouse RPTECs were extremely sensitive to reoxygenation, yet less sensitive to anoxia. These results indicate that although Syrian hamster drops body temperature during hibernation [28,29], its RPTECs are resistant to warm ischemia and reperfusion as well. Similar results in the kidney and other organs were obtained by other studies too [11,12,13].

Apoptotic cell death due to I-R injury was documented more than 25 years ago in rat renal tubules [30]; later, this finding has been confirmed by many studies [18,19]. We assessed cell apoptosis in RPTECs, by the level of activated cleaved caspase-3, in which both the intrinsic and the extrinsic apoptotic pathways converge [31]. Anoxia induced cell apoptosis in both human and mouse RPTECs, but not in hamster RPTECs. Reoxygenation induced cell apoptosis in human RPTECs, but not in mouse or hamster RPTECs. Regarding mouse RPTECs, more recent in vivo studies support that other types of cell death may play a more significant role than apoptosis in I-R injury. An elegant study showed that a necroptosis inhibitor protects mice from I-R-induced AKI, whereas inhibition of apoptosis with a pan-caspase inhibitor does not [32]. Later, the same group of investigators developed a new ferroptosis inhibitor, and indicate the pivotal role of this kind of cell death in I-R-induced AKI [33]. Concerning the resistance of hamster RPTECs to I-R-induced apoptosis, recent research has started to reveal the mechanisms by which mammalian hibernators escape from apoptosis due to I-R injury. Hibernators use selective gene expression to achieve inhibition of mitochondrial outer membrane permeabilization and prevent caspase activation, reversible protein phosphorylation to suppress metabolic rate and maintain ion homeostasis, and microRNA expression to inhibit pro-apoptotic signaling [34]. The possible clinical application of these findings remains to be evaluated. 

In parallel to apoptosis, the expression of the tumor suppressor p53, a known pro-apoptotic factor, was evaluated [35]. It should be noted that aside from apoptosis, p53 can also induce cell senescence. An elegant study using microarrays and chromatin immunoprecipitation showed that the more p53, the more the transcription of both proarrest and proapoptotic genes. To trigger apoptosis, cells must overcome an apoptotic threshold, whose height is determined by the expression levels of p53 and its targets, the duration of their expression, and the cellular context. Thus, above this threshold, cells are committed to apoptosis instead of senescence [36]. It is likely that certain conditions in our experiments overcome the apoptotic threshold leading to cell death. In all the evaluated species, under either anoxia or reoxygenation, alterations in the cleaved caspase-3 expression pattern fitted to the alterations of p53, suggesting the anoxia or reoxygenation-induced cell apoptosis may be p53-mediated. In accordance with our results, transient silencing of p53 with a specific siRNA protects renal function by attenuating apoptosis of RPTECs due to warm I-R injury in the rat kidney [37]. At present, two ongoing clinical studies aim to assess the efficacy and the safety of the p53-specific siRNA QPI-1002 in the context of ischemic AKI: one for preventing AKI following cardiac surgery (NCT02610283 phase 2), and the other for improving delayed graft function following kidney transplant in recipients of kidney from an older donor (NCT02610296 phase 3). 

Macroautophagy also characterizes AKI [20]. During macroautophagy (hereafter referred to as autophagy), portions of the cytoplasm are sequestrated within autophagosomes. Autophagosomes fuse with lysosomes, and the autophagic material is degraded. Besides its role in protein and organelle turnover, autophagy is upregulated under stress conditions, such as hypoxia and nutrient deprivation. In most cases, autophagy protects the cell from apoptotic death, especially if the level or the duration of stress is not lethal [38]. However, autophagy, per se, may also result in cell death [39]. In our model, autophagy, assessed by the LC3B-II to LC3B-I ratio [40], increased only in human RPTECs under either anoxia or reoxygenation. On the contrary, neither anoxia nor reoxygenation altered autophagy in mouse or hamster RPTECs. With regard to the role that autophagy may play in the death of human RPTECs due to anoxia or reoxygenation, this is unlikely to be the truth, since induction of autophagy in human RPTECs has been shown to delay cell deterioration due to anoxia, and to inhibit apoptosis [41]. The data about autophagy in hibernators are very limited. Concerning the absence of autophagy in mouse RPTECs under anoxia or reoxygenation, this may be influenced by our experimental conditions since, in another study, autophagy was observed in mouse RPTECs cultured under hypoxia, but not under anoxic conditions. In the last case, autophagy was detected only after several hours of reoxygenation. In the same study, the observed in vivo I-R-induced autophagy indicates the limitations of in vitro studies. Interestingly, in that study, autophagy proved renoprotective [42]. Similar results about the protective role of autophagy against AKI have been obtained by many other experimental studies [20], a fact that is against the deleterious role of autophagy in I-R injury. Also, all the above-described differences between human and the non-hibernator mouse RPTECs indicate that extrapolation of direct conclusions from mouse cells to phylogenetically distant human cells is not always valid.

Contrary to cell apoptosis, cell necrosis is characterized by the rupture of the cell membrane, and the release of the cellular content resulting in inflammation [43]. Until recently, it was thought that cell necrosis is an uncontrolled event, but recent research revealed that accidental cell death is caused only by very severe physical, chemical, or mechanical insults. As a consequence, accidental cell death is immediate and insensitive to pharmacologic interventions [43]. However, under most circumstances, cell death is regulated. Regulated cell death includes cell apoptosis and the various types of regulated cell necrosis, and involves a genetically encoded molecular machinery. Regulated cell death frequently occurs in a delayed manner, and is part of an adaptive response that unsuccessfully attempts to restore cellular homeostasis. Thus, like apoptosis, regulated cell necrosis can be altered using pharmacologic interventions which, from a clinical point of view, is very important [43]. 

Necroptosis, a type of regulated cell necrosis, has been described recently in I-R-induced AKI, and some studies claim that this is the main type of cell death that occurred in this situation [18,19]. However, in our model, necroptosis was not observed in any of the evaluated species, either under anoxia or reoxygenation. Necroptosis starts by the engagement of death receptors in the presence of caspase inhibitors, or by activation of receptors for pathogen and damage associated molecular patterns. Next, receptor-interacting protein kinases 1 and 3 (RIPK1/RIPK3) are activated, and phosphorylate MLKL, which multimerizes, translocates to the plasma membrane, and induces cell death [44]. The fact that we did not observe necroptosis due to anoxia or reoxygenation could be a result of the in vitro nature of our study. We cultured isolated RPTECs and, in the absence of immune cells and inflammation, not enough death receptor ligands are produced to trigger necroptosis. On the contrary, in in vivo experiments, immune cells are present, and inflammation plays a significant role in I-R-induced AKI [4]. It is likely that an initial I-R-induced type of cell necrosis releases danger-associated molecular patterns, which stimulate the immune cells and cause inflammation [45]. After that, the immune reaction may enhance the initial type or trigger additional types of cell death, such as apoptosis, necroptosis, or pyroptosis [18,19,43,44]. Thus, intervention to inhibit the initial I-R-induced type of regulated cell necrosis would also prevent a secondary, induced by an inflammation wave of various types of regulated cell death [43]. In accordance with our results, another study has shown that freshly isolated mouse tubules are not sensitive to spontaneous necroptosis, due to genetic ablation of either Fas-associated protein with death domain (FADD) or caspase-8, and that the RIPK1 inhibitor necrostatin-1 does not protect them from hypoxic injury [33]. 

Membrane lipid peroxidation by inducing cell membrane dysfunction causes cell death [21]. A relatively recently defined type of lipid peroxidation-induced cell death is ferroptosis [22]. Ferroptosis is a form of regulated cell necrosis that fits I-R injury, which is characterized by increased ROS production [1,2]. Ferroptosis was discovered by evaluating regulated cell necrosis induced by specific compounds. Targets of these compounds are the glutamate/cystine antiporter, which is required for the synthesis of the antioxidant glutathione, the labile iron pool, which is required for lipid peroxidation through the Fenton reaction, and glutathione peroxidase 4 (GPX4), which is the only enzyme that reduces lipid hydroperoxides within biological membranes. Initiation of cell membrane lipid peroxidation is followed by a propagation phase that is a chain reaction, during which one lipid peroxide provokes the oxidation of another lipid, and so on, resulting in severe cell membrane damage and necrosis. Interestingly, inhibition of ferroptosis protects mice from I-R-induced AKI, while hypoxia does not induce necroptosis in freshly isolated mouse tubules [33].

In our model, anoxia did not increase ROS production in any of the evaluated species, which is rational, since oxygen is required for ROS production. Reoxygenation increased ROS production considerably in cells from all the evaluated species. Regarding the hamster RPTECs, this indicates that the high levels of antioxidants, which have been detected in hibernating mammals [6,7], may be only partially protective. 

Under anoxia, lipid peroxidation, assessed by cellular MDA, remained unaffected in all evaluated species, which parallels the ROS levels that were unaltered by anoxia. Reoxygenation induced impressive lipid peroxidation in human and mouse RPTECs, while such an effect was not observed in hamster RPTECs, even though reoxygenation increased ROS in the last cells as well. Reasonably, lipid peroxidation-induced cell death takes place during reperfusion in human and mouse RPTECs, whereas hamster RPTECs escape lipid peroxidation-induced cell death. Considering the ferroptotic pathway, a well-defined pathway of lipid peroxidation-induced cell death [22], it is possible that hamster RPTECs may be protected by an increase in GPX4 activity or by a decrease in the cellular labile iron pool. To our knowledge, the cellular activity of GPX4 has not been evaluated yet in hibernating species. Regarding cellular iron, a recent study has shown that the primate hibernator *Cheirogaleus crossleyi* may be protected from iron-catalyzed oxidative damage by upregulating ferritin genes to store iron in a non-toxic form [46]. 

A limitation of our study is that we did not assess, thoroughly, the mechanisms that induce or protect from the various types of cell death in the evaluated species. However, our primary aim was to confirm the resistance to cell death due to warm anoxia or reoxygenation of RPTECs derived from the native hibernator Syrian hamster. Carrying on, by identifying the type of warm anoxia or reoxygenation-induced cell death in RPTECs in non-hibernating species, we set a starting point for further evaluation of the specific, and possibly clinically useful, pathways that are responsible for the escape of hamster cells from the types of cell death detected in human cells.

The in vitro nature of our study is also a limitation, since direct predictions from in vitro studies to the in vivo model are not always safe. However, as already noted, our study could be considered as a starting point for further investigation of the mechanisms that offer resistance to I-R injury in hibernators. For instance, recently, our team revealed significant differences in energy handling between RPTECs derived from the native hibernator Syrian hamster and the non-hibernator mouse [47]. Also, sometimes ex vivo experiments are required for clarifying the exact molecular mechanisms and the sequence of the events that lead to a certain end-point. In the cited study [33], the finding that, in isolated renal tubules, I-R induces ferroptosis but not necroptosis, which however is prevalent in the pathogenesis of I-R-induced AKI in vivo [32], may indicate that ferroptosis of RPTECs may be the initial insult, and the subsequent release of danger-associated molecular patterns and recruitment of immune cells trigger a second wave of cell death through necroptosis. As a parallel finding, the differences in the types of cell death, between human and mouse RPTECs, indicate that it is not always wise to extrapolate direct conclusions from the most commonly used experimental animal mouse to the phylogenetically distant human.

## 5. Conclusions

Our study indicates that RPTECs from the natural hibernator Syrian hamster resist cell death due to anoxia or reoxygenation, whereas human RPTECs die due to apoptosis and lipid peroxidation-induced cell death. Clarifying the ways that are responsible for the escape of hamster RPTECs from apoptosis and lipid peroxidation-induced cell death may lead to the development of interventions for preventing I-R-induced AKI in humans.

## Availability of Data and Materials

The analyzed datasets generated during the study are available from the corresponding author on reasonable request.
